# Thermodynamic characterization of the peptide assembly inhibitor binding to HIV-1 capsid protein

**DOI:** 10.1186/1742-4690-10-S1-P108

**Published:** 2013-10-11

**Authors:** Milan Kozisek, Jindrich Durcak, Jan Konvalinka

**Affiliations:** 1 Institute of Organic Chemistry and Biochemistry, Gilead Sciences and IOCB Research Center, Academy of Sciences of the Czech Republic, Prague, Czech Republic

## Background

The assembly of processed HIV-1 capsid protein into the mature core is essential step in viral cycle. Sticht et al. [1] identified a 12-mer peptide (CAI) that binds to a conserved hydrophobic groove of the C-terminal domain of capsid protein and inhibits virus assembly *in vitro.*

Here, we present results of detailed thermodynamic analysis of the CAI inhibitor binding to HIV-1 capsid protein and mutants thereof carrying mutations in the inhibitor binding pocket. The effect of the peptide on protein dimerization is determined and the minimal peptide sequence responsible for efficient inhibition is identified using microcalorimetry technique.

## Results

The isothermal titration calorimetry was used to examine the binding of CAI inhibitor to the wild-type capsid protein as well as the mutant variants. CAI binds to the capsid protein with favorable enthalpic contribution (-12.5 kcal.mol^-1^) and unfavorable entropic contribution (5.1 kcal.mol^-1^), and no protonation was observed during binding. The effect of several mutations inside the CAI binding pocket on dissociation constants determined by VP-ITC was similar as characterized by fluorescence polarization with fluorescently labeled CAI [2]. The thermodynamics of dissociation of capsid protein dimers in the absence and presence of CAI revealed that the peptide inhibitor did not have any significant effect on protein dimerization. The titrations with truncated variants of CAI peptide allowed us to determine that the minimal binding peptide consisted of 10 amino acids.

## Conclusions

The CAI binding site is very promising target for new antiretrovirotics. The detailed thermodynamic characterization of CAI binding to HIV-1 capsid protein performed within this study describes the complex formation and can help in further development of new HIV-1 assembly inhibitors.
